# Sub-2W tunable laser based on silicon photonics power amplifier

**DOI:** 10.1038/s41377-024-01681-1

**Published:** 2025-01-02

**Authors:** Neetesh Singh, Jan Lorenzen, Muharrem Kilinc, Kai Wang, Milan Sinobad, Henry Francis, Jose Carreira, Michael Geiselmann, Umit Demirbas, Mikhail Pergament, Sonia M. Garcia-Blanco, Franz X. Kärtner

**Affiliations:** 1 Center for Free-Electron Laser Science CFEL, Deutsches Elektronen-Synchrotron DESY, Hamburg, Germany; 2 Integrated Optical Systems, MESA+ Institute for Nanotechnology, University of Twente, 7500AE Enschede, The Netherlands; 3 LIGENTEC SA, EPFL Innovation Par L, Chemin de la Dent-d’Oche 1B, CH-1024 Ecublens, Switzerland; 4 Department of Physics, Universität Hamburg, Jungiusstr. 9, 20355 Hamburg, Germany

**Keywords:** Solid-state lasers, Photonic devices

## Abstract

High-power tunable lasers are intensely pursued due to their vast application potential such as in telecom, ranging, and molecular sensing. Integrated photonics, however, is usually considered not suitable for high-power applications mainly due to its small size which limits the energy storage capacity and, therefore, the output power. In the late 90s, to improve the beam quality and increase the stored energy, large-mode-area (LMA) fibers were introduced in which the optical mode area is substantially large. Such LMA fibers have transformed the high-power capability of fiber systems ever since. Introducing such an LMA technology at the chip-scale can play an equally disruptive role with high power signal generation from an integrated photonics system. To this end, in this work we demonstrate such a technology, and show a very high-power tunable laser with the help of a silicon photonics based LMA power amplifier. We show output power reaching 1.8 W over a tunability range of 60 nm, spanning from 1.83 µm to 1.89 µm, limited only by the seed laser. Such an integrated LMA device can be used to substantially increase the power of the existing integrated tunable lasers currently limited to a few tens of milliwatts. The power levels demonstrated here reach and surpass that of many benchtop systems which truly makes the silicon photonics based integrated LMA device poised towards mass deployment for high power applications without relying on benchtop systems.

## Introduction

High-power systems are indispensable for the next generation systems for commercial and scientific purposes. For example, coherent communication systems as well as frequency modulated detection and ranging applications require high power tunable lasers to increase the receiver and the target range, respectively^1–3^. Space agencies are interested in integrating high power lasers in miniaturized satellites for low-earth-orbit and deep space planetary observations^4–7^. Moreover, to handle the artificial intelligence driven heavy data center traffic, the emerging co-packaged optics technology requires integrated high-power lasers to satisfy the link budget^8,9^. Traditionally, high-power tunable lasers and amplifiers are considered to be in the realms of benchtop fiber and solid-state systems. Owing to their large system volume, they allow for large energy storage capacity and high gain saturation power and therefore high output signal power. In integrated photonics on the other hand, one of the main challenges is that it still lacks high-power lasers and amplifiers, which is mainly due to the tight modal confinement, that affords integrated photonics devices a small footprint and compact circuitry, but this fact comes into conflict with the large energy storage volume requirement and therefore the power of these lasers is usually limited. Even after decades of research, most of the integrated photonics demonstrations to date rely on benchtop fiber and solid-state lasers and amplifiers. Integrated semiconductor lasers and amplifiers are being used quite regularly in silicon photonics; however, they still face challenges with the low yield, high cost and complex heterogeneous integration. A bigger concern, however, is that the optical power from the semiconductor devices integrated to silicon photonics remain low, in the range of tens of mW^1,10–13^. This has caused researchers to explore ways to integrate high power semiconductor optical amplifiers (SOA)^14–18^, which work very well as standalone devices^19–22^, but their integration with silicon photonics has proven to be quite challenging. On the other hand, to repeat the success of rare-earth-doped (RED) fiber lasers and amplifiers, and to seamlessly integrate with fiber optic systems, integrated RED devices were explored in the last several decades^23,24^, as they have the potential for low noise and thermally insensitive signal generation compared to their semiconductor counterpart, a fact which was identified even before the invention of the fiber amplifiers (which also initiated research towards doping semiconductor materials with RE ions^25–27^). However, complex and compact circuitries were hard to achieve with such platforms and mass production seemed not possible which resulted in a gradual decline in research in those systems^23,24,28–33^. Over the last two decades, silicon-photonics based RED lasers and amplifiers have been explored^34–41^, where one has recently reached a power level up to 140 mW^42^ (albeit with a slightly complex fabrication process).

To reach power levels in integrated photonics similar to a table-top system, a device that can support large energy storage and high gain saturation power will be required. Large-mode-area (LMA) technology introduced in the late 90 s in fiber systems, thanks to the large optical mode, has substantially increased the stored energy and extractable power from fiber lasers and amplifiers^42,43^. Recently, we have shown a design of an integrated photonics based LMA waveguide^44^ which has been used for high energy pulse generation and pulse amplification^45–47^. In this work, we show a high-power continuous wave (CW) tunable laser based on silicon photonics high power LMA amplifier which increases the output power of the tunable laser up to 1.8 W. The device has a footprint of 4.5 mm^2^. The power level demonstrated surpasses that of many commercial benchtop lasers and is comparable to many benchtop amplifiers. Such an integrated LMA device can be used to substantially increase the power of the existing table top lasers as well as integrated photonics-based lasers where the power is limited to a few mWs to around 100 mW^1,10–13^. Especially, in the long wavelength window (up to 2 µm)—which has various medical, defense, and space applications, such as in laser surgeries (thanks to strong water absorption), greenhouse gas monitoring in earth and planetary space missions, and even in the next generation gravitational wave detection^5,48–54^—the power levels in integrated lasers as well as benchtop semiconductor lasers^19,55–57^ are severely limited. The integrated LMA technology can be used here not only to increase the power of such systems substantially, but it can also allow devices with lower cost, size, weight, and power (SWaP) that can be mass produced and replace many of their benchtop solid state and fiber counterparts.

## Results

**Fig. 1:**
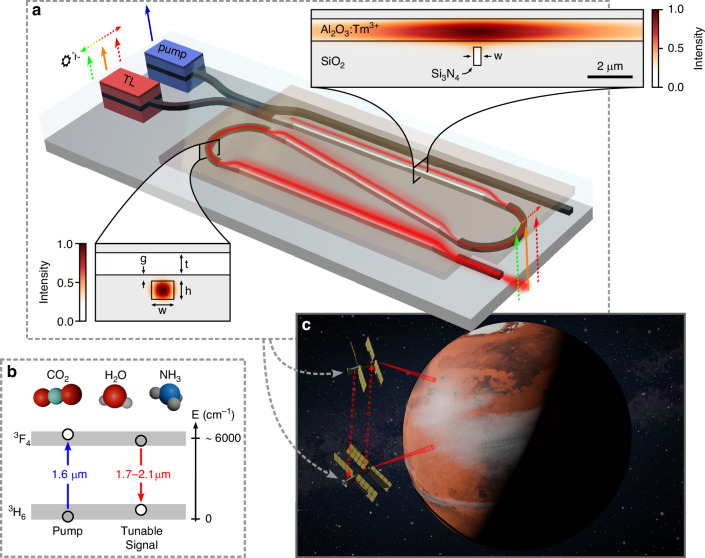
The device and a potential application. **a** A conceptual high-power tunable laser with an integrated high-power amplifier, pump, and seed tunable laser (TL). The amplifier has large mode area sections where, *w* = 280 nm, *t* = 1.35 µm and *h* = 800 nm. In the tight confinement region, the *w* = 1 µm. **b** A simplified energy diagram showing the pump and the signal levels. The signal gain bandwidth spans from 1.7 to 2.1 µm where the strong absorption lines of green-house gases and molecules essential for living organisms exist. **c** An artistic impression of a potential application of the high-power tunable laser where it is integrated to satellites for space applications, such as planetary exploration

The device is a hybrid device with an active medium on top of a passive layer^34,37,58–60^. It is fabricated in two steps for the passive and active parts. The passive part consists of a silicon substrate, 4 µm thick bottom oxide layer, 800 nm thick SiN layer and a 6.6 µm thick top oxide layer which is selectively etched down to 300 nm thickness to create an opening in the gain layer region. The gain waveguide cross-section is shown in Fig. 1a. Once the passive fabrication step is completed we coat the device with a gain layer. We use thulium-doped alumina gain film as it has a wide gain bandwidth ranging from 1.7 to 2.1 µm, which is important for molecular fingerprinting^61,62^, making it valuable for various medical, sensing, defense and space application. Additionally, due to such a large gain bandwidth high resolution LIDAR (with CW laser of wide tuning range) and ultra-short modelocked laser pulses can be realized. We deposited the thulium doped alumina gain layer (Tm^3+:^Al_2_O_3_) on a passive chip with radio frequency magnetron sputtering tool (details of the deposition process can be found in^63^). The targeted thulium concentration was 6 × 10^20^/cm^3^ and the film thickness was 1.35 µm. The device essentially consists of a strong mode confinement region and a weak mode confinement region. The strong mode confinement region which is based on SiN waveguides fully cladded in silica is for launching and collection of the pump and signal on and off the chip, and for facilitating compact bends to connect LMA gain sections, see the schematic in Fig. 1a. In the LMA sections the optical modes interact mainly with the gain-ion-doped alumina layer for high power signal generation. In the LMA, the calculated effective mode area of the pump mode is 33.5 µm^2^ and it ranges from 29 to 28 µm^2^ for the signal mode (from 1.8 to 1.95 µm). The optical mode confinement in the alumina layer ranges from 90 to 95% and 0.7 to 0.25% in the SiN layer over the wavelength range from 1.95 to 1.61 µm. The effective refractive index of the modes at the pump and around the signal wavelength window is 1.63 and ~1.61, respectively. The mode area is larger for the pump than the signal in the LMA section due the fact that the refractive index at the shorter wavelength is higher than that at longer wavelength, which causes the pump mode to be pulled up in the alumina layer more than the signal mode which results in a slightly larger pump mode. The weak mode overlap with the SiN layer helps to reduce the scattering loss due to SiN sidewalls and therefore the loss is mainly defined by the alumina film loss. The LMA sections are connected to each other with adiabatic tapers and circular bends. The measured taper loss is around 0.05–0.1 dB/taper and the bend loss is between 0.003 and 0.008 dB/180° (see Supplementary).

**Fig. 2:**
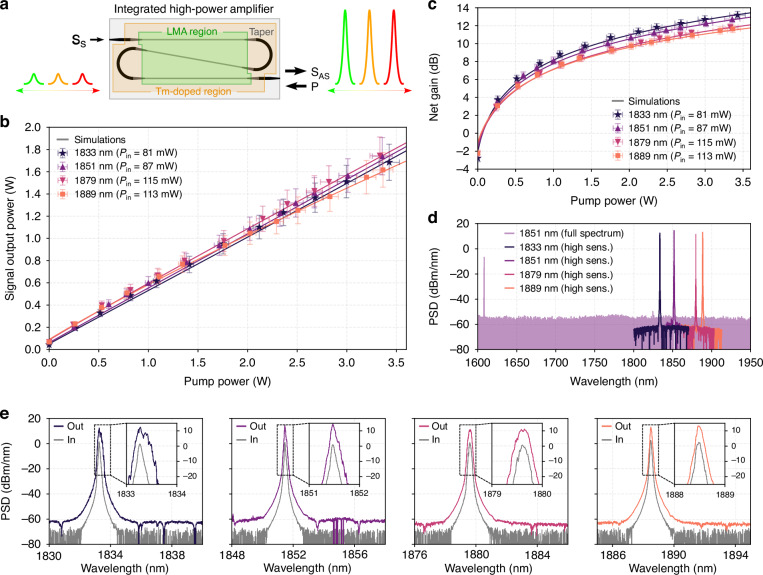
High power output results. **a** A schematic of the counter propagating scheme where the pump is launched from the opposite direction than the tunable seed laser. The seed tunable laser Ss is amplified to high power (S_AS_) through the LMA amplifier (see Supplementary for details of the experimental setup). **b** Amplified signal output power of the seed laser as a function of coupled pump power for signal tuned from 1.83 to 1.89 µm with on-chip seed input power ranging from 81 mW to 115 mW. **c** Net gain as a function of pump power. The error-bars include an uncertainty in coupling power of 0.3 dB, especially at high output power. **d** Broadband optical spectra taken of the amplified seed laser show the presence of negligible ASE pedestal between the pump and the signal (which is mainly due to the use of high-power seed source). **e** Optical spectra of the amplified output (taken for a net gain of 10–12 dB) and the input signal from 1830 nm to 1890 nm

The pump and tunable seed lasers are launched into the chip as shown in Fig. 2a (see Supplementary for the details of the setup). Due to the lack of a suitable commercial tunable laser, for the experiment we used a home-built laser^64^ which could be tuned from 1830 nm to 1890 nm. Fine tunability can be achieved with an intracavity narrow bandpass filter and by purging the cavity of water vapor present in air. The fiber-to-chip coupling loss of the pump and the signal to the device were roughly 2.2 and 2.4 dB/facet respectively. We measured the device in counterpropagating pump configuration (pump and signal from the opposite side of the chip), however, the copropagating pumping (pump and signal from the same side of the chip) gives the same results. This is due to the fact that the amplified spontaneous emission (ASE), which usually takes away the gain from a weak signal (in small signal gain testing), is dominated by the stronger seed signal used here^65^. We obtained the maximum gain when the pump and signal were launched in the same polarization. The polarization extinction ratio was measured before and after the chip to be >30 dB. The input and output polarization of the signal and pump after propagating through the chip were measured to be the same, showing no signs of polarization conversion. We tested devices with several lengths, of which the 6 cm long device produced the highest power. The seed wavelength range was from 1833 to 1889 nm limited only by the tunability of the home-built laser. To demonstrate the high-power signal generation and high gain saturation power capability of the LMA device, we launched the maximum seed power possible from our tunable laser which ranged from 80-115 mW, which is also close to the maximum output power of tunable lasers in integrated photonics^1^, allowing us to demonstrate the readiness of the LMA device for full integration with the best existing integrated tunable lasers for high power amplification. The experimental results are shown in Fig. 2b–e. The measured amplified output power is between 1650 and 1800 mW, corresponding to a net gain of ~11.5–13.5 dB. Such high gains for high power seed lasers were only possible with table top fiber-based power amplifiers or semiconductor amplifiers before. Even higher gain, up to 16–17 dB is achievable, beyond which parasitic lasing from the facet reflections happens^41,42,66^. Such parasitic lasing, (a) in a free space system can be circumvented with high power index matching glues at the facets or angled input/output couplers (for example in booster amplification), and (b) will not be present in a fully integrated systems where LMA amplifiers will be seamlessly integrated with integrated tunable lasers. The pump power required for the high-power signal generation was higher than expected, which was due to a slightly higher passive film loss (0.25 dB/cm @ 1.85 µm) which can be reduced with an optimized film deposition to be below 0.1 dB/cm which is routinely obtained with RF sputtering^67^. The experimental results match well with the simulation performed with a set of steady state rate equations (see Supplementary). A wide bandwidth spectrum is shown in Fig. 2d spanning from the pump wavelength to 1950 nm. Here we see no signs of ASE pedestal (lower than 70 dB) for the signal amplified around 1850 nm at high power which demonstrates a clean amplification of the signal (here the OSA, Yokogawa AQ6376, measures only a fraction of the total output power, see supplementary). The measured optical spectrum of the signal at the input and the output is shown in Fig. 2e. Here, we observe that the signal to noise ratio is high after amplification (up to 70 dB) and a slight broadening of the spectrum happens, which is mainly due to the feedback resulting from the reflection of the amplified signal from the output facet of the chip back to the seed laser, which can be avoided as mentioned above. We also tested longer devices, for example, an 11 cm and a 15 cm long device was measured, giving 0.5 dB and 0.8 dB lower gain than the 6 cm device. At higher pump powers, however, the gain will be higher for longer devices simply because of a higher total number of ions that the signal can interact with. Furthermore, we tested the stability of the device, and it showed negligible variation in performance over several hours of testing.

We measured the noise figure (NF) of the amplifier as a function of gain, seed power and wavelength. The NF is obtained by using the relation,NF = [P_ASE_/(hυΔυG)] + 1/G^65^. Here, P_ASE_ is the ASE power measured over 0.5 nm bandwidth (Δυ) with the OSA, hυ is the signal photon energy and G is the linear gain (ratio of amplified signal output to input seed power).

**Fig. 3:**
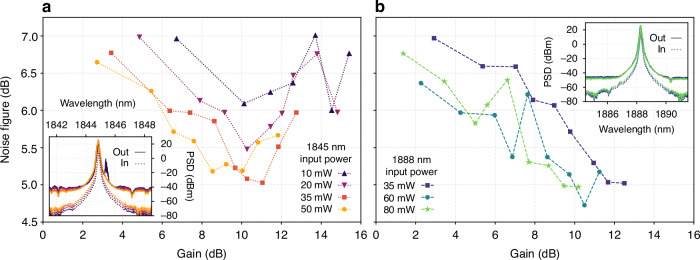
Noise figure results. **a**, **b** Noise figure as a function of gain at 1845 nm and 1888 nm for different on chip seed power, respectively. The insets show the input and amplified output signal spectrum (a parasitic lasing spike is seen close to 1845 nm with 10 mW seed when the net gain reaches close to 16 dB)

To get the correct estimate of the ASE power, P_ASE_ is measured in the spectral window very close to the signal wavelength (~4 nm away from the signal). We chose two wavelengths of the seed laser for the NF measurement, namely 1845 nm and 1888 nm. This is because the laser remained most stable at these wavelengths during the entire NF measurement, and since they are very close to the other tested wavelengths, 1830 nm and 1890 nm, the data is representative of the entire tunable laser bandwidth. The experimental data is shown in Fig. 3 where the gain and NF are in logarithmic scale. The signal gain for all the measurements was varied with the pump power. The NF vs gain for different seed power at 1845 nm and 1888 nm are shown in Fig. 3a, b. The NF reduces with gain by 1–2 dB over the measured range. The NF is high at low gain because it is inversely proportional to gain and due to weak inversion of the gain ions (due to weaker pumping), which increases the spontaneous emission factor (n_sp_) which is directly proportional to the ASE power, P_ASE_~n_sp_^65^. The NF is lower for higher seed power, which is also expected simply because the seed with higher power dominates over the ASE in pulling down the ions from the upper state compared to weaker seed signals. The NF is almost the same for the two wavelengths (1845 and 1888 nm) for similar seed powers.

**Fig. 4:**
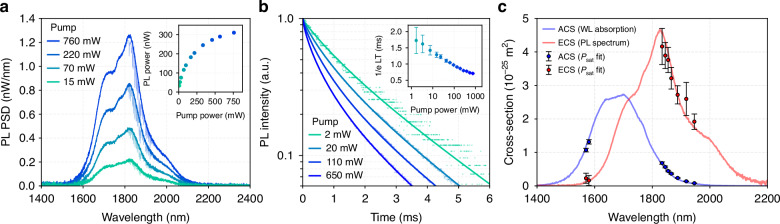
Spectroscopic measurements. **a** The PL spectra at different pump power. Inset shows the integrated PL power vs pump power. **b** PL time domain trace with respect to pump power. Inset shows 1/e lifetime (the time it takes for the signal to drop by 63.2% from its peak). The error bars include standard deviation of ten recorded traces. **c** The measured and extrapolated ECS (*σ*_em_, emission cross-section) and ACS (*σ*_abs_, absorption cross-section) of the gain film. The error bars include uncertainties in the gain-ion concentration and the mode area of ~5–10%

Furthermore, we measured the luminescence properties of the Tm^3+^ ions. In the photoluminescence (PL) measurements, to avoid effects such as, reabsorption, ASE and wavelength dependent loss in the waveguide we measured the luminescence with an out-of-plane setup (see Supplementary). In this scheme, the pump is launched into the chip with end-fire coupling; however, the light from the excited ions is collected from the top of the chip. The measured spectral and time domain responses are shown in Fig. 4a, b. The shape of the PL spectra shows negligible dependence on the pump power, and the total power of the PL increases with the pump power. This increase in PL power is due to the high saturation power afforded by the LMA design.

The 1/e lifetime reduces with the pump power from around 1.8 ms to 0.7 ms mainly due to energy transfer up-conversion of ions. In that, the excited ions at the ^3^F_4_ level transfer their energy (non-radiatively) to neighboring excited ions, raising them to the ^3^H_4_ state (1.58 eV) and thus effectively increasing the non-radiative decay rate from the ^3^F_4_ level causing a reduction in the overall fluorescence lifetime. We fit the lifetime curves with Zubenko’s model^68^ and extracted the intrinsic (fluorescence) lifetime to be 2.5 ms, which is dependent on the radiative and phonon assisted non-radiative lifetime. Absolute emission and absorption cross-section values (*σ*_em_ and *σ*_abs_) of the active material were determined from intrinsic saturation power measurements (^69^, see Supplementary) which were applied to Füchtbauer-Ladenburg relation to extract the radiative lifetime to be 4.2 ms. The measured *σ*_em_ and *σ*_abs_ are shown in Fig. 4c.

**Fig. 5:**
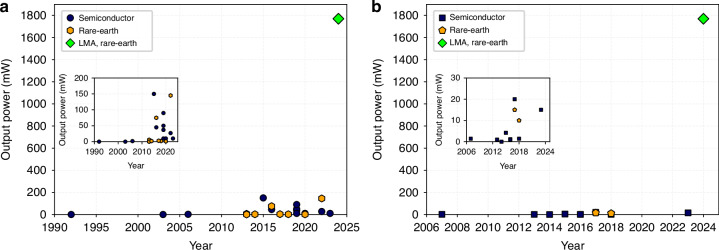
Comparison of techonolgies, and the timeline of the development of lasers and amplifiers. **a** The output power from silicon photonics devices (independent of wavelength of operation) since the 90s^34,37,38,41,57–59,70,74–79^. The semiconductor here represent hybrid and hetrogenously integrated lasers and amplifiers on silicon platform. The rare-earth devices include mainly sputtering and ion implantation based rare-earth doped silicon photonics amplifiers and lasers. LMA, rare-earth is the device shown in this work. The inset shows zoomed-in graph. **b** The output power from a silicon photonics device around 1.9 µm since 2007 compared to the rare-earth doped silicon photonics devices^80–87^ and the LMA device shown in this work. Inset shows zoomed-in graph clearly showing the the power levels of the lasers and amplifiers based on other technologies

We plot the output power from silicon photonics based integrated lasers and amplifiers demonstrated in the last few decades (Fig. 5). Here we can see how the power has increased from the early works of ref. ^70^ on hybrid integration of semiconductor lasers on silicon chip. The rare-earth devices integrated onto silicon photonics platform have seen a growth in the output power since around 2013–2015 and have reached beyond 100 mW recently^41^. Similar growth pattern is seen for the devices operating at longer wavelength around 2 µm (Fig. 5b). In comparison, our work has significantly improved the output power from a silicon photonics device, regardless of the wavelength of operation, making it comparable to many benchtop systems.

## Discussion

In conclusion, we have demonstrated a very high-power tunable laser based on integrated silicon photonics based LMA amplifier with output power reaching close to 1.8 W. Tunability from 1830 nm to 1890 nm has been shown, with the potential for high power amplification even over a broader bandwidth (to exploite the wider gain window of thulium gain system). Such a device can substantially increase the power of not only an off-chip tunable laser, as shown in this work, but also an on-chip tunable laser such as the ones based on semiconductor or rare-earth gain which are currently limited to within 1 to 100 mW of output power. Achieving that will significantly simplify the system as the light from an integrated tunable laser can be seamlessly launched into an LMA amplifier without suffering from parasitic Fresnel reflections (as is the case with the off-chip tunable lasers). The measured optical-to-optical and importantly the electrical-to-optical conversion efficiency (wall-plug efficiency) can be significantly improved with the co-integration of the high-power pump diode, which is currently limited by the off-chip pump laser (see Supplementary). For application requiring high small-signal gain (high gain for a weak input signal), the limitation imposed by the parasitic lasing due to facet reflections can be avoided by anti-reflection coating at the chip facet or by employing high power index matching fluid, allowing one to achieve net gain beyond 30 dB. Such a high power, fully integrated amplifier will find various applications, for example, in medical surgeries where currently big systems based on holmium and thulium lasers are used^52^. The LMA high power lasers can significantly reduce the system size and cost allowing large scale deployment. Such systems can be implemented not only for terrestrial air pollution control, for example, harmful gas emission from combustion engine^71–73^, but space agencies are highly interested in integrated photonics-based devices especially with watt level capability for space communications and projects where cost-effective observation of greenhouse gases in the atmosphere with high precision using small satellites is a major challenge. Moreover, molecules important for biological processes such as ammonia and water can be explored in outer space for signs of life with the miniature satellites and space probes installed with high power integrated lasers. Next generation gravitation wave detectors will benefit from miniature high-power lasers operating around 1.9 µm, which can be used with cryogenically cooled silicon mirrors replacing silica mirrors to reduce thermal and quantum noise, extending the horizon of gravitation wave detection^48^. Such devices can be used not only in earth observatories, but can also play a key role in space-based gravitation wave detectors where the system-size reduction is of utmost importance. Moreover, such devices can be scaled to other wavelength windows, such as the high-power laser window at 1 µm and the telecom window around 1.3 µm and 1.55 µm. Therefore, we believe such a silicon photonics device as a stand-alone unit can replace many fiber-based tunable lasers and amplifiers with the potential for mass production, elevating the integrated photonics application landscape to unprecedented levels.

## Supplementary information

Supplementary Information

## Data Availability

The data that supports the plots within this paper and other findings of this study are available from the corresponding author upon reasonable request.

## References

[1] Kobayashi, N. et al. Silicon photonic hybrid ring-filter external cavity wavelength tunable lasers. *J. Lightwave Technol.* **33**, 1241–1246 (2015).

[2] Canoglu, E. et al. Semiconductor lasers and optical amplifiers for LiDAR photonic integrated circuits. *Proc. 27th International Semiconductor Laser Conference* 1–2 (IEEE, 2021).

[3] Singh, N. et al. Silicon photonics optical frequency synthesizer. *Laser Photonics Rev.* **14**, 1900449 (2020).

[4] Graydon, O. Conquering the final frontier. *Nat. Photonics* **12**, 647–648 (2018).

[5] Storm, M., Cao, H., Albert, M. & Engin, D. Cubesat lidar concepts for ranging, topology, sample capture, surface, and atmospheric science. In *Proceedings of the Small Satellite Conference*, Logan, UT, USA, 5–10 August 2017; Paper SSC17-S2-03.

[6] Dailey, J. M. et al. High output power laser transmitter for high-efficiency deep-space optical communications. *Proceedings of SPIE 10910, Free-Space Laser Communications XXXI* 109100M (SPIE, 2019).

[7] Krainak, M. et al. Integrated photonics for NASA applications. *Proceedings of SPIE 10899, Components and Packaging for Laser Systems V* 108990F (SPIE, 2019).

[8] Tan, M. et al. Co-packaged optics (CPO): status, challenges, and solutions. *Front. Optoelectron.* **16**, 1 (2023). 36939942 10.1007/s12200-022-00055-y PMC10027985

[9] Zhou, W. J. et al. High power CW laser for co-packaged optics. *Conference on Lasers and Electro-Optics* 1–2 (IEEE, 2022).

[10] Huang, D. N. et al. High-power sub-kHz linewidth lasers fully integrated on silicon. *Optica* **6**, 745–752 (2019).

[11] Guo, Y. Y. et al. Hybrid integrated external cavity laser with a 172-nm tuning range. *APL Photonics* **7**, 066101 (2022).

[12] Van Rees, A. et al. Ring resonator enhanced mode-hop-free wavelength tuning of an integrated extended-cavity laser. *Opt. Express* **28**, 5669–5683 (2020). 32121783 10.1364/OE.386356

[13] Soltanian, E. et al. Micro-transfer-printed narrow-linewidth III-V-on-Si double laser structure with a combined 110 nm tuning range. *Opt. Express* **30**, 39329–39339 (2022). 36298887 10.1364/OE.470497

[14] Kharas, D. et al. High power (>300 mW) 1550 nm on-chip laser realized using passively aligned hybrid integration. *Conference on Lasers and Electro-Optics* 1–2 (IEEE, 2020).

[15] Gao, Y. K. et al. High-power, narrow-linewidth, miniaturized silicon photonic tunable laser with accurate frequency control. *J. Lightwave Technol.* **38**, 265–271 (2020).

[16] Chen, C. et al. Hybrid integrated Si_3_N_4_ external cavity laser with high power and narrow linewidth. *Opt. Express* **31**, 26078–26091 (2023). 37710477 10.1364/OE.487850

[17] McKinzie, K. A. et al. InP high power monolithically integrated widely tunable laser and SOA array for hybrid integration. *Opt. Express* **29**, 3490–3502 (2021). 33770946 10.1364/OE.413434

[18] Zeng, S. W. et al. Watt-level beam combined diode laser systems in a chip-scale hybrid photonic platform. *Opt. Express* **30**, 23815–23827 (2022). 36225055 10.1364/OE.461877

[19] Juodawlkis, P. W. et al. High-power, low-noise 1.5-μm slab-coupled optical waveguide (SCOW) emitters: physics, devices, and applications. *IEEE J. Sel. Top. Quantum Electron.* **17**, 1698–1714 (2011).

[20] Smith, G. M. et al. Slab-coupled optical waveguide lasers and amplifiers. *Proceedings of SPIE 8241, High-Power Diode Laser Technology and Applications X* 82410S (SPIE, 2012).

[21] Leisher, P. O. et al. >3W diffraction-limited 1550nm diode laser amplifiers for LIDAR. *Proceedings of SPIE 11982, Components and Packaging for Laser Systems VIII* 1198206 (SPIE, 2022).

[22] Campbell, J. et al. 30dBm single mode fiber-coupled semiconductor optical amplifier at 1550 nm. *28th International Semiconductor Laser Conference* 1–2 (IEEE, 2022).

[23] Hibino, Y. et al. Neodymium-doped silica optical waveguide laser on silicon substrate. *IEEE Photonics Technol. Lett.* **1**, 349–350 (1989).

[24] Aoki, H., Maruyama, O. & Asahara, Y. Glass waveguide laser. *IEEE Photonics Technol. Lett.* **2**, 459–460 (1990).

[25] Mears, R. J. et al. Low-noise erbium-doped fibre amplifier operating at 1.54μm. *Electron. Lett.* **23**, 1026–1028 (1987).

[26] Ennen, H. et al. 1.54-µm luminescence of erbium-implanted III-V semiconductors and silicon. *Appl. Phys. Lett.* **43**, 943–945 (1983).

[27] Tsang, W. T. & Logan, R. A. Observation of enhanced single longitudinal mode operation in 1.5-µm GaInAsP erbium-doped semiconductor injection laser. *Appl. Phys. Lett.* **49**, 1686–1688 (1986).

[28] Lallier, E. Rare-earth-doped glass and LiNbO_3_ waveguide lasers and optical amplifiers. *Appl. Opt.* **31**, 5276–5282 (1992). 20733706 10.1364/AO.31.005276

[29] Lallier, E. et al. Laser oscillation of single-mode channel waveguide in Nd: MgO: LiNbO_3_. *Electron. Lett.* **25**, 1491–1492 (1989).

[30] Mackenzie, J. I. Dielectric solid-state planar waveguide lasers: a review. *IEEE J. Sel. Top. Quantum Electron.* **13**, 626–637 (2007).

[31] Grivas, C. Optically pumped planar waveguide lasers, Part I: fundamentals and fabrication techniques. *Prog. Quantum Electron.* **35**, 159–239 (2011).

[32] Ter-Gabrielyan, N. et al. Resonantly pumped single-mode channel waveguide Er: YAG laser with nearly quantum defect limited efficiency. *Opt. Lett.* **38**, 2431–2433 (2013). 23939071 10.1364/OL.38.002431

[33] Van Dalfsen, K. et al. Thulium channel waveguide laser with 1.6 W of output power and ∼80% slope efficiency. *Opt. Lett.* **39**, 4380–4383 (2014). 25078182 10.1364/OL.39.004380

[34] Purnawirman et al. C- and L-band erbium-doped waveguide lasers with wafer-scale silicon nitride cavities. *Opt. Lett.* **38**, 1760–1762 (2013). 23862218 10.1364/ol.38.001760

[35] Li, N. X. et al. Monolithically integrated erbium-doped tunable laser on a CMOS-compatible silicon photonics platform. *Opt. Express* **26**, 16200–16211 (2018). 30119455 10.1364/OE.26.016200

[36] Agazzi, L. et al. Monolithic integration of erbium-doped amplifiers with silicon-on-insulator waveguides. *Opt. Express* **18**, 27703–27711 (2010). 21197045 10.1364/OE.18.027703

[37] Belt, M. et al. Arrayed narrow linewidth erbium-doped waveguide-distributed feedback lasers on an ultra-low-loss silicon-nitride platform. *Opt. Lett.* **38**, 4825–4828 (2013). 24322142 10.1364/OL.38.004825

[38] Magden, E. S. et al. Monolithically-integrated distributed feedback laser compatible with CMOS processing. *Opt. Express* **25**, 18058–18065 (2017). 28789295 10.1364/OE.25.018058

[39] Rönn, J. et al. Ultra-high on-chip optical gain in erbium-based hybrid slot waveguides. *Nat. Commun.* **10**, 432 (2019). 30683870 10.1038/s41467-019-08369-w PMC6347631

[40] Bradley, J. D. B. et al. Monolithic erbium- and ytterbium-doped microring lasers on silicon chips. *Opt. Express* **22**, 12226–12237 (2014). 25051584 10.1364/oe.22.012226

[41] Liu, Y. et al. A photonic integrated circuit-based erbium-doped amplifier. *Science* **376**, 1309–1313 (2022). 35709288 10.1126/science.abo2631

[42] Ranaud, C. C. et al. Characteristics of Q-switched cladding-pumped ytterbium-doped fiber lasers with different high-energy fiber designs. *IEEE J. Quantum Electron.* **37**, 199–206 (2001).

[43] Richardson, D. J., Nilsson, J. & Clarkson, W. A. High power fiber lasers: current status and future perspectives [Invited]. *J. Opt. Soc. Am. B* **27**, B63–B92 (2010).

[44] Singh, N., Ippen, E. & Kärtner, F. X. Towards CW modelocked laser on chip—a large mode area and NLI for stretched pulse mode locking. *Opt. Express* **28**, 22562–22579 (2020). 32752515 10.1364/OE.396703

[45] Singh, N. et al. Silicon photonics-based high-energy passively *Q*-switched laser. *Nat. Photonics* **18**, 485–491 (2024).

[46] Singh, N. et al. Watt-class CMOS-compatible power amplifier. *Conference on Lasers and Electro-Optics Europe & European Quantum Electronics Conference* 1 (IEEE, 2023).

[47] Gaafar, M. A. et al. Femtosecond pulse amplification on a chip. *Nat. Commun.* **15**, 8109 (2024). 39285172 10.1038/s41467-024-52057-3 PMC11405508

[48] Kapasi, D. P. et al. Tunable narrow-linewidth laser at 2 μm wavelength for gravitational wave detector research. *Opt. Express* **28**, 3280–3288 (2020). 32122000 10.1364/OE.383685

[49] Fried, N. M. & Irby, P. B. Advances in laser technology and fibre-optic delivery systems in lithotripsy. *Nat. Rev. Urol.* **15**, 563–573 (2018). 29884804 10.1038/s41585-018-0035-8

[50] Hodgkinson, J. & Tatam, R. P. Optical gas sensing: a review. *Meas. Sci. Technol.* **24**, 012004 (2013).

[51] Li, Z. et al. Thulium-doped fiber amplifier for optical communications at 2 µm. *Opt. Express* **21**, 9289–9297 (2013). 23609639 10.1364/OE.21.009289

[52] Scholle, K. et al. 2µm laser sources and their possible applications. in *Frontiers in Guided Wave Optics and Optoelectronics* (ed Pal, B.) (IntechOpen, 2010).

[53] Baker, W. E. et al. Lidar-measured winds from space: a key component for weather and climate prediction. *Bull. Am. Meteorol. Soc.* **76**, 869–888 (1995).

[54] Schliesser, A., Picqué, N. & Hänsch, T. W. Mid-infrared frequency combs. *Nat. Photonics* **6**, 440–449 (2012).

[55] Zia, N. et al. Widely tunable 2 µm hybrid laser using GaSb semiconductor optical amplifiers and a Si_3_N_4_ photonics integrated reflector. *Opt. Lett.* **48**, 1319–1322 (2023). 36857278 10.1364/OL.480867

[56] Wang, R. J. et al. Widely tunable 2.3 µm III-V-on-silicon Vernier lasers for broadband spectroscopic sensing. *Photonics Res.* **6**, 858–866 (2018).

[57] Volet, N. et al. Semiconductor optical amplifiers at 2.0-µm wavelength on silicon. *Laser Photonics Rev.* **11**, 1600165 (2017).

[58] Choi, S. J. et al. Microdisk lasers vertically coupled to output waveguides. *IEEE Photonics Technol. Lett.* **15**, 1330–1332 (2003).

[59] Fang, A. W. et al. Electrically pumped hybrid AlGaInAs-silicon evanescent laser. *Opt. Express* **14**, 9203–9210 (2006). 19529301 10.1364/oe.14.009203

[60] Chen, H. W., Kuo, Y. H. & Bowers, J. E. A hybrid silicon-AlGaInAs phase modulator. *IEEE Photonics Technol. Lett.* **20**, 1920–1922 (2008).

[61] Bremer, K. et al. Sensitive detection of CO_2_ implementing tunable thulium-doped all-fiber laser. *Appl. Opt.* **52**, 3957–3963 (2013). 23759843 10.1364/AO.52.003957

[62] Gordon, I. E. et al. The HITRAN2020 molecular spectroscopic database. *J. Quant. Spectrosc. Radiat. Transf.* **277**, 107949 (2022).

[63] Van Emmerik, C. I. et al. Relative oxidation state of the target as guideline for depositing optical quality RF reactive magnetron sputtered Al_2_O_3_ layers. *Optical Mater. Express* **10**, 1451–1462 (2020).

[64] Demirbas, U. et al. Continuous-wave Tm:YLF laser with ultrabroad tuning (1772-2145 nm). *Opt. Express* **30**, 41219–41239 (2022). 36366605 10.1364/OE.471288

[65] Becker, P. C., Olsson, N. A. & Simpson, J. R. *Erbium-Doped Fiber Amplifiers* (Academic Press, 1999).

[66] Brown, D. C., Jacobs, S. D. & Nee, N. Parasitic oscillations, absorption, stored energy density and heat density in active-mirror and disk amplifiers. *Appl. Opt.* **17**, 211–224 (1978). 20174386 10.1364/AO.17.000211

[67] Worhoff, K. et al. Reliable low-cost fabrication of low-loss Al_2_O_3_: Er^3+^ waveguides with 5.4-dB optical gain. *IEEE J. Quantum Electron.* **45**, 454–461 (2009).

[68] Zubenko, D. A. et al. Different mechanisms of nonlinear quenching of luminescence. *Phys. Rev. B* **55**, 8881–8886 (1997).

[69] Saleh, A. A. M. et al. Modeling of gain in erbium-doped fiber amplifiers. *IEEE Photonics Technol. Lett.* **2**, 714–717 (1990).

[70] Friedrich, E. E. L. et al. Hybrid integration of semiconductor lasers with Si-based single-mode ridge waveguides. *J. Lightwave Technol.* **10**, 336–340 (1992).

[71] Sandström, L. et al. Gas monitoring using semiconductor lasers operating in the 2 *µ*m wavelength region. *Infrared Phys. Technol.* **39**, 69–75 (1998).

[72] Sonnenfroh, D. M. & Allen, M. G. Observation of CO and CO_2_ absorption near 1.57 µm with an external-cavity diode laser. *Appl. Opt.* **36**, 3298–3300 (1997). 18253339 10.1364/ao.36.003298

[73] Ikeda, Y. et al. A sensor for measuring CO_2_ gas temperature and concentration using 2µm DFB semiconductor laser. *Proc. of 10th International Symposium on Laser Techniques for Fluid Mechanics* 483–485 (Springer, 2002).

[74] Zhao, H. W. et al. High-power indium phosphide photonic integrated circuits. *IEEE J. Sel. Top. Quantum Electron.* **25**, 4500410 (2019). 10.1109/JSTQE.2018.2866677 PMC6219397 30416332

[75] Van Gasse, K., Wang, R. J. & Roelkens, G. 27dB gain III-V-on-silicon semiconductor optical amplifier with >17dBm output power. *Opt. Express* **27**, 293–302 (2019). 30645375 10.1364/OE.27.000293

[76] Hosseini, E. S. et al. CMOS-compatible 75 mW erbium-doped distributed feedback laser. *Opt. Lett.* **39**, 3106–3109 (2014). 24875988 10.1364/OL.39.003106

[77] Davenport, M. L. et al. Heterogeneous silicon/III–V semiconductor optical amplifiers. *IEEE J. Sel. Top. Quantum Electron.* **22**, 3100111 (2016).

[78] Matsumoto, T. et al. Hybrid-integration of SOA on silicon photonics platform based on flip-chip bonding. *J. Lightwave Technol.* **37**, 307–313 (2019).

[79] Wang, Y. et al. Efficiency-boosted semiconductor optical amplifiers via mode-division multiplexing. *Optica* **10**, 1153–1160 (2023).

[80] Dong, P. et al. 1.9µm hybrid silicon/III-V semiconductor laser. *Electron. Lett.* **49**, 664–666 (2013).

[81] Roelkens, G. et al. Silicon-based photonic integration beyond the telecommunication wavelength range. *IEEE J. Sel. Top. Quantum Electron.* **20**, 394–404 (2014).

[82] Wang, R. J. et al. 2.3µm range InP-based type–II quantum well Fabry-Perot lasers heterogeneously integrated on a silicon photonic integrated circuit. *Opt. Express* **24**, 21081–21089 (2016). 27607711 10.1364/OE.24.021081

[83] Boehm, G. et al. Growth of InAs-containing quantum wells for InP-based VCSELs emitting at 2.3 µm. *J. Cryst. Growth* **301-302**, 941–944 (2007).

[84] Wang, R. J. et al. Heterogeneously integrated III-V-on-silicon 2.3x *µ*m distributed feedback lasers based on a type-II active region. *Appl. Phys. Lett.* **109**, 221111 (2016).

[85] Spott, A. et al. Heterogeneously integrated 2.0 µm CW hybrid silicon lasers at room temperature. *Opt. Lett.* **40**, 1480–1483 (2015). 25831364 10.1364/OL.40.001480

[86] Shtyrkova, K. et al. Integrated CMOS-compatible Q-switched mode-locked lasers at 1900nm with an on-chip artificial saturable absorber. *Opt. Express* **27**, 3542–3556 (2019). 30732372 10.1364/OE.27.003542

[87] Li, N. X. et al. Broadband 2-µm emission on silicon chips: monolithically integrated holmium lasers. *Opt. Express* **26**, 2220–2230 (2018). 29401762 10.1364/OE.26.002220

