# Supplementary material for: Sub-2W tunable laser based on silicon photonics power amplifier
Source: Light Sci Appl. 2025 Jan 2;14:18. doi: 10.1038/s41377-024-01681-1 (PMC11693756; doi:10.1038/s41377-024-01681-1)
Supplement: Supplementary file 1 — Supplementary Information [file 41377_2024_1681_MOESM1_ESM.pdf]

Supplementary Information for "Sub-2W tunable laser based on silicon photonics power amplifier"

Neetesh Singh[1*], Jan Lorenzen[1], Muharrem Kilinc[1], Kai Wang[2], Milan Sinobad[1], Henry Francis[3], Jose Carriera[3], Michael Geiselmann[3], Umit Demirbas[1], Mikhail Pergament[1], Sonia M Garcia-Blanco[2], and Franz X. Kärtner[1,4]

[1]*Center for Free-Electron Laser Science CFEL, Deutsches Elektronen-Synchrotron DESY, Germany*
[2]*Integrated Optical Systems, MESA+ Institute for Nanotechnology, University of Twente, 7500AE, Enschede, The Netherlands*
[3]*LIGENTEC SA, EPFL Innovation Par L, Chemin de la Dent-d'Oche 1B, Switzerland CH-1024 Ecublens, Switzerland*
[4]*Department of Physics, Universität Hamburg, Jungiusstr. 9, 20355 Hamburg, Germany*
**neetesh.singh@desy.de*

Setup and measurement method:

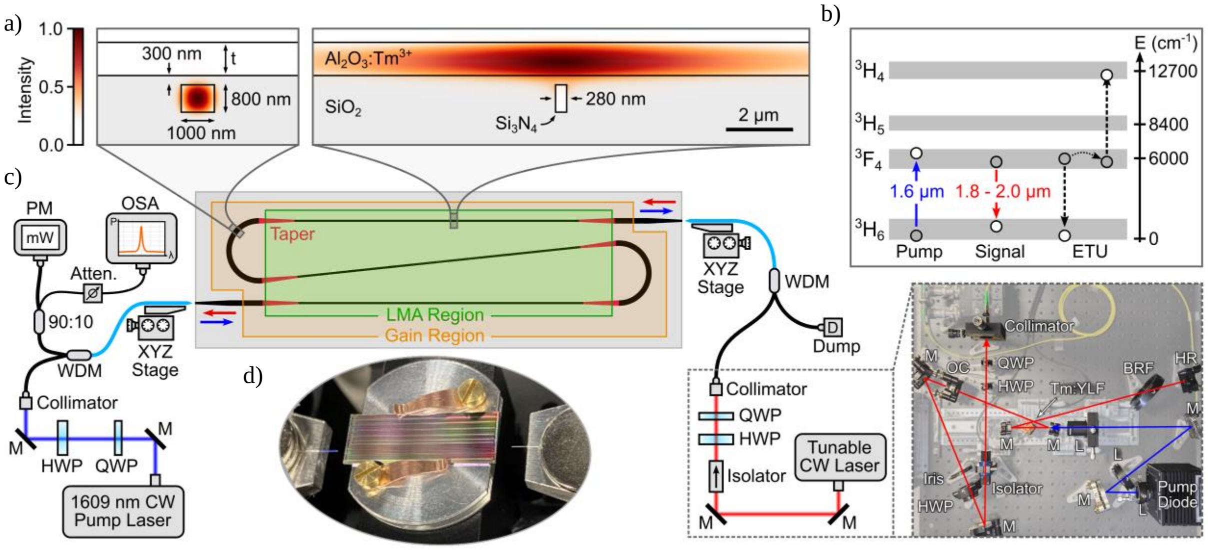

Figure S1. The experimental setup. a) The small and the large mode area regions on the chip are connected to each other with tapers. b) A simplified energy diagram of $Tm^{3+}:Al_2O_3$. ETU, energy transfer up-conversion. c) The pump and tunable laser coupling to the chip are shown. M, mirror; HWP, half wave plate; QWP, quarter wave plate; WDM, wavelength division multiplexer (pump and signal combiner); PM, power meter; Atten. is a variable attenuator; XYZ stage is a three-axis flexure translation stage; Dump ports are for dumping the residual pump. A picture of the tunable laser is shown on the signal side, where, a collimator is used to couple the laser into a fibre. L, Lens; OC, output coupler; and Tm:YLF is the gain crystal. The blue line indicates the pump path and the red line indicates the signal path. d) A picture of the chip mounted on a heat conducting plate.

The details of the counterpropagating pumping setup for high-power signal generation from a tunable laser and the power amplifier based on the LMA waveguide are shown in Fig. S1. In this, the high power LMA amplifier chip was mounted on a flat aluminum plate and locked in place with copper clips and brass screws allowing high thermal conductivity for high power testing. The chip mount is placed on a blank device mount (*Thorlab* HBB002). Due to the lack of availability of high-power tunable laser in the desired wavelength range, for the experiment, we have used a home-built linear cavity Tm:YLF solid-state tunable laser (Fig. S1) [1]. A 7 mm long 3% Tm-doped Brewster-cut YLF crystal is pumped by a 792 nm single-emitter multimode diode (*RPMC* LDX-3315-792-C). The maximum pump optical power used is 3.5 W. A 2 mm thick crystal quartz birefringent filter (BRF) with an off-surface optical axis is inserted into the cavity at Brewster's angle for tuning the Tm:YLF laser output wavelength. The BRF has a diving angle of 25°, and provides a free-spectral range of around 600 nm. The TE-polarized part of the intracavity beam undergoes Fresnel reflection losses from the laser crystal and the BRF plate surfaces, which grants a sinusoidally varying spectral transmission response with a modulation depth of around 45%. The Tm:YLF crystal currently employed is optimized for lasing in the E//c axis due to the higher absorption and emission cross section of this axis compared to E//a. However, the emission dip of the E//c axis around 1850 nm deteriorates the tuning behavior in this region. In future work, the E//a axis of Tm:YLF could be employed for smoother tuning near 1850 nm. Moreover, a multi-plate BRF configuration or a narrow bandpass interferometric filter could also be used to increase the modulation depth and finesse of the filter for improved sideband rejection during tuning. An isolator (*Faraday Photonics*) is placed at the output of the laser with an isolation strength ranging from 20-30 dB between 1800 nm to 1900 nm, having the peak isolation at 1860 nm. An isolator in the setup is required, as the reflections from the chip facets cause instability in the tunable laser causing high fluctuation in the wavelength

and laser power. By purging the cavity of water vapor present in air, and as mentioned above, by implementing a high finesse intracavity filter and multiple isolators at the output (to give 60 to 90 dB feedback suppression) one can achieve a wider and fine-step wavelength tunability. We must emphasize, however, that these issues will not be present in an all integrated tunable laser as a seed source [2]. The pump is an amplified low noise CW laser at 1.61 µm (*Alnair labs*, TLG 220, amplified with an *IPG* amplifier, EAR-10-1610-LP-SF). As shown in Fig. S1c, the pump passes through a set of waveplates for polarization control followed by a free-space to fiber-coupler (collimator) where it is coupled to a WDM. The WDMs on both sides (in the contra-directional pumping) are spliced to 3 µm spot size lensed fibers that have an insertion loss of 1.5 to 2.5 dB over the wavelength range from 1.8 to 1.9 µm. At the pump side (which is the output side of the signal), the signal port of the WDM is connected to a 90:10 splitter (*Thorlabs* TW2000R2A1B which splits the power into 90 and 10 %). The 90% port goes into a calibrated power meter (*Thorlab* S148C, PM100D). The 10% goes through a variable attenuator to an optical spectrum analyzer (OSA, *Yokogawa* AQ6376, set at 0.05nm resolution). The OSA measures approximately 23 dB lower power than the power meter when considering the attenuator, 10% tap, WDM insertion loss and the connector losses. The polarization was fixed at the output of the lensed fibers by replacing the chip with a sequence of a linear grid polarizer and a beam collimator which allowed only desired polarization (controlled by waveplates) to pass through. After the polarization alignment, without touching any fiber the chip is brought into coupling position where the lights are coupled with the lensed fibers. One can also determine the effectiveness of this setup by estimating the optical-to-optical and electrical-to-optical conversion efficiencies achieved with this setup. At the maximum amplified on-chip signal power of ~ 1.8 W (output fiber approx. 1 W), see Fig. 2b in the main manuscript, the on-chip pump power is around 3.3 W (power in input fiber approx. 5.5 W). This results in on-chip optical conversion efficiency, which is given as ($Ps_{(amp/onchip)}$ – $Ps_{(in/onchip)}$)/$Pp_{(onchip)}$, to be approx. 51%. Here the $Ps_{(amp/onchip)}$, $Ps_{(in/onchip)}$, and $Pp_{(onchip)}$ are the on-chip amplified signal power, input signal power and pump power, respectively. Since the input and output couplers are not optimized, the off-chip (fiber to fiber) conversion efficiency is lower, close to 15%. With the optimized couplers, which would allow 1 dB coupling loss (as is usually available on a silicon nitride platform [3]), the off-chip conversion efficiency (fiber to fiber) can be >30% which can be increased even further up to 50% when the LMA waveguide design is optimized and passive losses are further reduced for higher optical to optical conversion efficiency on-chip (which can reach more than 80% for the thulium gain system [4]). Moreover, the wall plug efficiency (electrical to optical) can be improved, which is quite low currently (<1%) due to using a commercial benchtop pump laser (with only ~ 6% wall plug efficiency), by directly pumping the device with a multimode pump diode that can have more than 70% wall plug efficiency [5, 6]. Such pump diodes can be integrated either with the well-developed technique of micro optical bench or by edge coupling [7, 8]. Hence, an optimally designed device for high conversion efficiency together with the right pump diode can allow ~ 35% of wall plug efficiency.

Gain simulation: The high-power signal amplification was simulated with a set of modified stead-state rate equations for in band pumping as given below [9, 10]:

$$\frac{dN_2}{dt} = W_{ETU}N_{1,(a,q)}^2 - \frac{1}{\tau_2}N_{2,(a,q)} = 0 \qquad (1)$$

$$\frac{dN_{1,(a,q)}}{dt} = R_{P,(a,q)} - R_{S,(a,q)} - 2W_{ETU}N_{1,(a,q)}^2 + \frac{1}{\tau_2}N_{2,(a,q)} - \frac{1}{\tau_1}N_{1,(a,q)} = 0 \qquad (2)$$

$$f_{(a,q)}N_d = N_{0,(a,q)} + N_{1,(a,q)} + N_{2,(a,q)} \qquad (3)$$

$$R_{P,(a,q)} = \frac{\lambda_P}{hc}I_P\left[\sigma_{a,P}N_{0,(a,q)} - \sigma_{e,P}N_{1,(a,q)}\right] \qquad (4)$$

$$R_{S,(a,q)} = \frac{\lambda_S}{hc}I_S\left[\sigma_{e,S}N_{1,(a,q)} - \sigma_{a,S}N_{0,(a,q)}\right] \qquad (5)$$

The population density is denoted by N_i where i = 0, 1 or 2 represent the ground state, $^3H_6$, upper state (amplifier state) $^3F_4$, and the up-conversion higher state, $^3H_4$. The excitation of ions from $^3F_4$ to $^3H_4$ state increases with the pump power which in turn reduces the lifetime of the ions at the upper state. The high concentration device is simulated with 7% quenched ions and the low concentration device is simulated with 5% of the quenched ions. The fraction of active and quenched ions is given as $f_{(a,q)}$ where, 'a' and 'q' represent active and quenched ions, respectively. The lifetime (τ_2) of the $^3H_4$ level is considered to be in the range of 1-30 µs, and the intrinsic lifetime (τ_1) of the $^3F_4$ is measured for the low concentration and the high concentration film to be 2.8 ms and 2.5 ms, respectively. The measurement method is discussed in the next section. The pumping of the ions to upper state (level 1) is dependent on the pump rate, R_P, and the signal amplification is dependent on the signal generation rate,

R_s. Here, the I_P and I_s are the pump and signal intensity, respectively. The doping concentration, N_d, is ~6 x10^{20}/cm^3 for high concentration film and 4x10^{20}/cm^3 for the low concentration film. The absorption and emission cross-sections parameters are given as σ_a and σ_e, respectively. The energy-transfer-up-conversion (ETU) in rare-earth ions is a non-linear process, requiring the interaction between two excited ions, hence the term N_1^2 in equation 2. The proportionality factor, $W_{ETU} = {\pi^2}/{3}\sqrt{C_{DD}C_{DA}}N_d$, where the parameters accounting for the energy transfer between donor and donor, and donor and acceptor are given by C_{DD} and C_{DA}, respectively. Values for C_{DD} and C_{DA} are estimated from the absorption and emission cross-section overlap and the fit parameters for the excited state lifetime with Zubenko's model [11] to be around 5.1 x 10^{-51} m^6/s and 1.3 x 10^{-52} m^6/s, respectively. The signal amplification as a function of the pump power can be estimated by the following relations:

$$\frac{dP_p(z)}{dz} = P_P(z)\left\{\iint \psi_P\,[\sigma_{e,P}N_{1,(a,q)} - \sigma_{a,P}N_{0,(a,q)})]dxdy - \alpha_{loss,P}\right\} \qquad (7)$$

$$\frac{dP_s(z)}{dz} = P_s(z)\left\{\iint \psi_s[\sigma_{e,S}N_{1,(a,q)} - \sigma_{a,S}N_{0,(a,q)}]dxdy - \alpha_{loss,S}\right\} \qquad (8)$$

Here, the overlap of pump and signal mode with the gain is described by ψ_P and ψ_s. The signal and pump mode area are simulated to be around 30 µm^2 using Lumerical mode solver. The propagation loss is given as $\alpha_{loss,P}$ and $\alpha_{loss,S}$ for the pump and signal light, respectively.

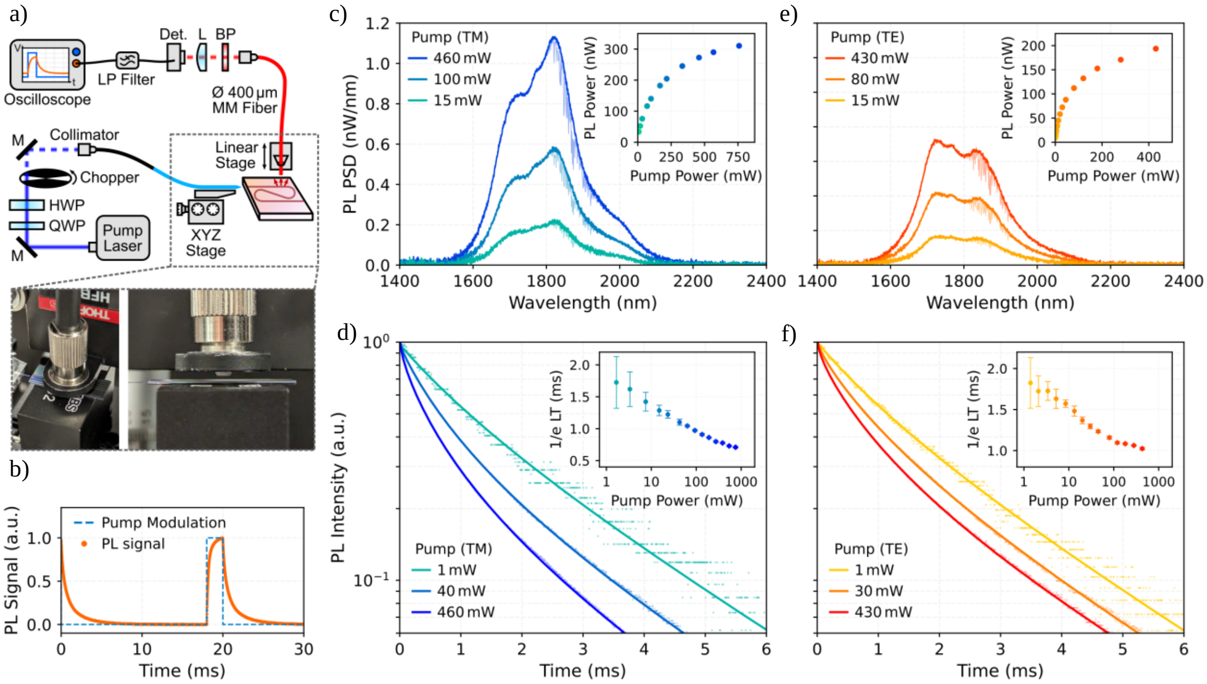

Figure S2. a) PL setup. LP filter – 20 KHz low pass filter. L-lens, BP- bandpass filter, MM-multimode fiber, HWP – half wave plate, QWP-quarter wave plate, M-mirror. B) Pump modulation and the resulting PL signal generation in time. c) and d) PL spectral and time domain trace of the high concentration chip taken at different pump power. Inset shows integrated PL power with respect to pump power. e) and f) are the PL spectral and time domain trace for low concentration device for different pump power. Inset shows pump power dependent 1/e lifetime.

Spectroscopic properties of the gain film: We studied the luminescence properties of the Tm^{3+} ions in the device using a photoluminescence (PL) setup as shown in Fig. S2a. In order to avoid effects such as, reabsorption, ASE and wavelength dependent loss in the waveguide we measured the luminescence with an out-of-plane setup. Essentially the pump is launched into the chip using end-fire coupling and the light from the excited ions is collected from the top of the chip. The details are as follows. The pump laser is chopped and is coupled into the amplifier waveguide with a lensed fiber. A multimode fiber (400 µm core *Thorlabs* M124L02) was aligned right above the chip to collect the fluorescence as shown in Fig. S2a. The collected light is either sent directly in to an OSA for spectral measurement or launched into another free-space setup to filter out the scattered pump with a bandpass filter (*Thorlabs* FB2000-500) and then focused onto an extended InGaAs detector (*Thorlabs* PDA10D2)

for lifetime measurement. High frequency noise in the detector signal is filtered out by a 20 kHz low noise lowpass filter (*Thorlabs* EF122) which is subsequently connected to a digital oscilloscope (*RS* pro RSDS 1304CFL). The time domain trace is taken on the oscilloscope which was triggered by the chopper controller (Fig. S2b). The spectral and time domain data were taken at multiple locations along the length of the amplifiers; however, very weak spectral variation and negligible temporal variations were observed. The fluorescence strength varied by 10% for a mm change in the vertical position of the collection fiber. The measured spectral and time domain responses are shown in Fig. S2c-f for a low (~ 4 $x10^{20}/cm^3$) and a high gain ion concentration chip (~ 6 $x10^{20}/cm^3$). The low concentration chip was deposited with the gain layer after 6 months of the high concentration chip. The PL spectral shape show negligible dependence on the pump power for both of the chips. However, we do see a difference in the shape of PL spectra from the two chips. The high concentration chip shows a strong peak around 1830 nm and the low concentration chip shows relatively flatter profile from 1720 to 1880 nm. Such differences are usually seen in RF sputtered films and are mostly dependent on the film morphology which can vary from device to device. These variations are undesirable and are currently being investigated to be avoided in future. The integrated PL power as a function of pump power is shown in the inset. The PL power increases with the pump power as larger numbers of ions are excited as the power increases; moreover, the PL saturation seems to be beyond the pump power levels investigate here. In a conventional waveguide amplifier and fiber amplifier the PL power usually saturates at a lower power [12], that is mainly because the dopants are confined either in a smaller rectangular region in a waveguide, or a circular region in a fiber. In an LMA device used here, the dopants are confined spatially only in the vertical direction but not in the *horizontal* direction. That is because the gain layer is essentially an unetched thin film. Therefore, at high pump power the pump photons, in a highly asymmetric LMA mode, keep exciting the gain ions along the horizontal direction (further away from the center). This results in very high-power PL saturation which also helps in increasing the gain saturation power even further (in addition to the increase due to large mode area). The 1/e lifetime (the time it takes for the PL power to drop by 63.2%) reduces for the high concentration chip from 1.5 ms to 0.7 ms and for the low concentration chip it varies from 1.8 ms to 1 ms for the pump power ranging from 1 mW to 1 W. The drop in the 1/e lifetime with the pump power is due to the energy transfer up-conversion of ions. In that, the excited ions at $^3F_4$ level transfer their energy (non-radiatively) to neighboring excited ions, raising them to $^3H_4$ state and thus effectively increasing the non-radiative decay rate from the $^3F_4$ level which effectively causes a shorter lifetime. We fit the lifetime curves with Zubenko's model [11] and extract the fluorescence lifetime, also known as intrinsic or upper-state lifetime (which is dependent on the radiative and phonon assisted non-radiative lifetime), for the two chips to be 2.5 ms (high concentration) and 2.8 ms (low concentration).

The emission and absorption cross-sections (shown in Fig. 3 in the main text) were extracted by measuring the saturable loss of the gain waveguide [13] which is highly dependent on the spectroscopic properties of the medium. The details will be shown elsewhere [14], here we briefly describe the method. Absolute emission and absorption cross-section values (σ_{em} and σ_{abs}) of the active material were determined from intrinsic saturation power measurements, as the saturation power $P_{sat} = A_{eff} / [\Gamma(\sigma_{em}+\sigma_{abs})\ \tau]$, where A_{eff} is the effective mode area and τ is the ETU-dependent excited state lifetime (1/e lifetime) and Γ is the mode overlap with the active layer. With this method we can extract both σ_{abs} and P_{sat} [13]. In that, the power-dependent transmission of a 11 cm long amplifier device was measured at two pump wavelengths (1.57 and 1.58 µm, with Alnair labs, TLG 220) and various signal wavelengths (1.83 - 1.94 µm, with the home-built tunable Tm:YLF laser). A two-level amplifier model is fit to the transmission data to extract the amplifiers saturation power, from which the cross-section values are obtained as free fit parameters. We confirm the obtained values of σ_{abs} by measuring independently σ_{abs} over a broad band using the loss spectrum of the waveguide obtained with a low power white light source (where the waveguide loss is related to the σ_{abs}, the active ion concentration and mode overlap to the active region). Once the σ_{abs} and P_{sat} are obtained the σ_{em} is extracted using the relation of saturation power given above. After obtaining the σ_{abs} and σ_{em}, using the Füchtbauer-Ladenburg relation one can obtain an accurate value of the radiative lifetime of the gain ions, which is around 4.2 ms and 4.5 ms for the high and low concentration gain films, respectively. There are other methods to extract the spectroscopic parameters; however, only with a limited accuracy. For example, McCumber theory requires the knowledge of partition functions and Stark level splitting which are not known for $Tm^{3+}:Al_2O_3$ amorphous films; and the Judd-Ofelt theory can only be applied (to obtain the radiative life time) if transition cross-sections of several transitions (well below 800 nm) can be measured, which is currently not possible with our devices.

References

1. U. Demirbas, et. al. "Continuous-wave Tm:YLF laser with ultrabroad tuning (1772-2145 nm)," Opt. Express 30 (2022).

2. N. Kobayashi et. al. “Silicon Photonic Hybrid Ring-Filter External Cavity Wavelength Tunable Lasers,” J. Light wave technol. 2015.
3. G. Brunetti et. al. “Silicon nitride spot size converter with very low-loss over the C-Band,” IEEE, Photonics Tech. Lett. 35 (2023).
4. A. Sincore et. al., “High average power thulium-doped silica fiber lasers: review of systems and concepts,” *IEEE J. Sel. Top*. 24 (2018).
5. P. Crump et. al. “85% power conversion efficiency 975-nm broad area diode lasers at − 50°C, 76 % at 10°C,” CLEO JWB24 (2006).
6. B. Wang et. al. “71% wall-plug efficiency from 780 nm-emitting laser diode with GaAsP quantum well,” Optics and Laser Technol. 168 (2024).
7. B. Snyder, et. al. “Hybrid integration of the wavelength-tunable laser with a silicon photonic integrated circuit,” J. Light Wave Technology, 31 (2013).
8. M. Theurer et. al., “Flip-chip integration of InP to Sin photonic integrated circuits,” *J. Light. Technol.* 38 (2020).
9. S. A. V. Cordova et. al. “Erbium-doped spiral amplifiers with 20 dB of net gain on silicon,” Opt. Express, 22 (2014).
10. L. Agazzi, "Spectroscopic excitation and quenching processes in rare-earth-ion-doped Al2O3 and their impact on amplifier and laser performance", Thesis (2012).
11. D. A. Zubenko et. al. “Different mechanisms of nonlinear quenching of luminescence”, Phys. Rev. B 55, 8881-8886 (1997).
12. S. D. Agger and J. H. Povlsen. Emission and absorption cross-section of thulium doped silica fibers. Opt. Express, 14, 2006.
13. A. A. M. Saleh et. al. “Modeling of gain in erbium-doped fiber amplifiers,” IEEE Photonics Technology Letters, 2 (1990).
14. J. Lorenzen et. al. “Spectroscopic properties of thulium doped high power integrated LMA amplifiers,” CLEO US 2024
